# Tumor suppressive microRNA-1285 regulates novel molecular targets: Aberrant expression and functional significance in renal cell carcinoma

**DOI:** 10.18632/oncotarget.417

**Published:** 2012-01-30

**Authors:** Hideo Hidaka, Naohiko Seki, Hirofumi Yoshino, Takeshi Yamasaki, Yasutoshi Yamada, Nijiro Nohata, Miki Fuse, Masayuki Nakagawa, Hideki Enokida

**Affiliations:** ^1^ Department of Urology, Graduate School of Medical and Dental Sciences, Kagoshima University, Kagoshima, Japan; ^2^ Department of Functional Genomics, Graduate School of Medicine, Chiba University, Chiba, Japan

**Keywords:** microRNA, expression signature, miR-1285, tumor suppressor, renal cell carcinoma, transglutaminase 2

## Abstract

MicroRNAs (miRNA) are non-coding RNAs, approximately 22 nucleotides in length, which function as post-transcriptional regulators. A large body of evidence indicates that miRNAs regulate the expression of cancer-related genes involved in proliferation, migration, invasion, and metastasis. The aim of this study was to identify novel cancer networks in renal cell carcinoma (RCC) based on miRNA expression signatures obtained from RCC clinical specimens. Expression signatures revealed that 103 miRNAs were significantly downregulated (< 0.5-fold change) in RCC specimens. Functional screening (cell proliferation assays) was performed to identify tumor suppressive activities of 20 downregulated miRNAs. Restoration of mature miRNAs in cancer cells showed that 14 miRNAs (*miR-1285*, *miR-206*, *miR-1*, *miR-135a*, *miR-429*, *miR-200c, miR-1291, miR-133b, miR-508-3p, miR-360-3p, miR-509-5p, miR-218, miR-335, miR-1255b* and *miR-1285*) markedly inhibited cancer cell proliferation, suggesting that these miRNAs were candidate tumor suppressive miRNAs in RCC. We focused on *miR-1285* because it significantly inhibited cancer cell proliferation, invasion, and migration following its transfection. We addressed *miR-1285*-regulated cancer networks by using genome-wide gene expression analysis and bioinformatics. The data showed that transglutaminase 2 (*TGM2*) was directly regulated by *miR-1285*. Silencing of the target gene demonstrated significant inhibition of cell proliferation and invasion in the RCC cells. Furthermore, immunohistochemistry showed that TGM2 expression levels in RCC specimens were significantly higher than those in normal renal tissues. Downregulation of tumor suppressive *miR-1285*, which targets oncogenic genes including TGM2, might contribute to RCC development. Thus, *miR-1285* modulates a novel molecular target and provides new insights into potential mechanisms of RCC oncogenesis.

## INTRODUCTION

Renal cell carcinoma (RCC) is the most common neoplasm of the adult kidney, and clear cell RCC represents the most common renal cancer histology [1]. Despite increased early detection of RCC and more frequent surgery, the mortality rate has not changed significantly during the last two decades [2, 3]. New therapeutic drugs have been developed for treatment of metastatic RCC. However it is difficult to treat patients with metastatic RCC and prognostic improvement cannot be achieved. Therefore, it is crucial to find novel molecular mechanisms based on recent genome-wide studies including non-coding RNAs (ncRNA) in RCC oncogenesis and metastasis.

RNA can be divided into two categories, protein coding RNA and ncRNA. It is important to examine the functions of ncRNAs and their association with human disease, including cancer. microRNAs (miRNAs) are endogenous small ncRNA molecules (~19 - 22 bases) that regulate protein coding gene expression by repressing translation or cleaving RNA transcripts in a sequence-specific manner [4]. A growing body of evidence suggests that miRNAs are aberrantly expressed in many human cancers, and that they play significant roles in their initiation, development, and metastasis [5]. Some highly expressed miRNAs could function as oncogenes by repressing tumor suppressors, whereas low level miRNAs could function as tumor suppressors by negatively regulating oncogenes [6].

Genome-wide miRNA expression signatures can rapidly and precisely reveal aberrant expression of miRNA in cancers. Thus, we have conducted miRNA expression signature analyses and searched for tumor suppressive miRNAs in various types of cancers [7-9]. Our previous studies successfully identified several tumor suppressive miRNAs such as *miR-1*, *miR-133a*, *miR-145*, *miR-489*, *miR-218*, *miR-375* and *miR-874* [8-13].

The aim of this study was to identify new tumor suppressive miRNAs revealed in our expression signature analyses of clinical RCC specimens. We focused on *miR-1285*, which had the greatest inhibitory effect on cell proliferation in our functional analysis. We also used genome-wide gene expression analysis to search for novel targets regulated by *miR-1285* in RCC cells. Our data showed that 11 genes had putative target sites for *miR-1285* in their 3'-untranslated regions (3'UTR). Tumor suppressive *miR-1285* mediates novel molecular targets and provides new insights into the potential mechanisms of RCC oncogenesis.

## RESULTS

### Identification of downregulated miRNAs in RCC: assessment of miRNA expression signatures

We evaluated mature miRNA expression levels of clinical RCC specimens (ten cancer tissues and five adjacent non cancerous tissues) by miRNA expression signature analysis. Expression signatures revealed that 103 miRNAs were downregulated (< 0.5-fold change) in RCC specimens (Supplementary Table 1). The top 20 miRNAs (*miR-141*, *miR-200c*, *miR-187*, *miR-509-5p*, *miR-135a*, *miR-508-3p*, *miR-1285*, *miR-206*, *miR-218*, *miR-133b*, *miR-1291*, *miR-let-7g*, *miR-204*, *miR-429*, *miR-370*, *miR-363*, *miR-335*, *miR-1*, *miR-1255B* and *miR-362-3p*) in the expression list were subjected to further study (Table 1).

**Table 1:** Down-regulated microRNAs in renal cell carcinoma (RCC)

microRNA	P-value	Normal	Cancer	Fold Change (Cancer/Normal)
hsa-miR-141	0.022	1.237	0.026	0.021
hsa-miR-200c	0.022	1.104	0.024	0.021
hsa-miR-187	0.007	1.526	0.043	0.028
hsa-miR-509-5p	0.003	1.196	0.050	0.042
hsa-miR-135a	0.003	1.525	0.099	0.065
hsa-miR-508-3p	0.007	1.321	0.096	0.072
hsa-miR-1285	0.020	1.777	0.171	0.096
hsa-miR-206	0.013	1.580	0.192	0.121
hsa-miR-218	0.005	1.506	0.197	0.130
hsa-miR-133b	0.006	1.173	0.173	0.147
hsa-miR-1291	0.019	1.978	0.310	0.157
hsa-let-7g*	0.031	1.508	0.247	0.164
hsa-miR-204	0.014	1.468	0.254	0.173
hsa-miR-429	0.003	1.267	0.222	0.175
hsa-miR-370	0.042	1.525	0.268	0.176
hsa-miR-363	0.010	1.377	0.244	0.177
hsa-miR-335	0.005	1.226	0.224	0.182
hsa-miR-1	0.005	1.017	0.189	0.186
hsa-miR-1255B	0.020	1.306	0.248	0.190
hsa-miR-362-3p	0.010	1.501	0.312	0.208

### Transfection of 20 downregulated miRNAs: effects on cancer cell proliferation

**Figure 1:**
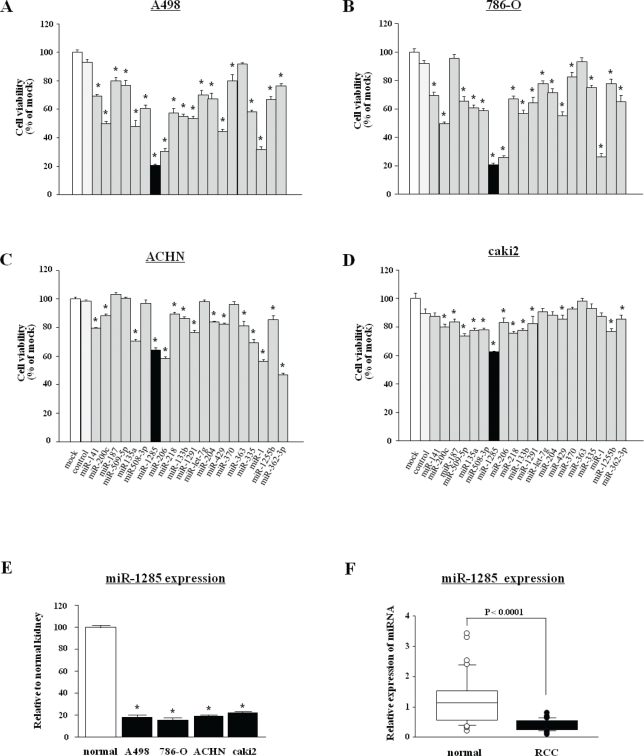
Screening of tumor suppressive microRNAs in RCC (A-D) Effect of cell proliferation determined by XTT assays using mature miRNAs (*miR-141*, *miR-200c*, *miR-187*, *miR-509-5p*, miR-135a, miR-508-3p, miR-1285, miR-206, miR-218, miR-133b, miR-1291, let-7g*, *miR-204, miR-429, miR-370, miR-363, miR-335, miR-1, miR-1255b*, and *miR-362-3p*) after 72 h transfection of RCC cell lines (A498, 786-O, ACHN and caki2). *P < 0.0001. (E) Expression levels of *miR-1285* in RCC cell lines. *miR-1285* expression levels were significantly downregulated in all cell lines in comparison with normal kidney. (F) Expression levels of *miR-1285* in clinical RCC specimens. Relative *miR-1285* expression levels are expressed in box plots. *RNU48* was used as the internal control.

To investigate the functional role of the 20 downregulated miRNAs, we performed gain-of-function studies using mature miRNA transfection in RCC cell lines. The XTT assay revealed significant inhibition of cell proliferation in several miRNA transfectants (A498, 786-O, ACHN and caki2) in comparison with mock transfectants (transfectant reagent only) (each, P < 0.0001, Figure 1A, B, C, and D). Supplementary Table 2 shows the extent to which cell proliferation was inhibited in four RCC cell lines. *miR-1285* transfection showed the greatest inhibitory effect among the 20 candidate miRNAs. Thus, we focused on *miR-1285* and investigated the functional significance using RCC cell lines.

### Expression levels of *miR-1285* in cancer cell lines and RCC clinical specimens

The expression levels of *miR-1285* were significantly lower in RCC cell lines (A498, 786-O, ACHN and caki2) than normal kidney (Figure 1E). Also, *miR-1285* expression was significantly reduced in RCC clinical specimens compared with adjacent non-cancerous tissues (P < 0.0001, Figure 1F).

### Effect of *miR-1285* restoration on cell proliferation, migration and invasion in RCC cell lines

To investigate the functional significance of *miR-1285*, we performed gain-of-function studies using transient transfection with mature *miR-1285*. We utilized two sources of mature *miR-1285* (Ambion and Thermo) to ensure reproducibility of the data.

**Figure 2:**
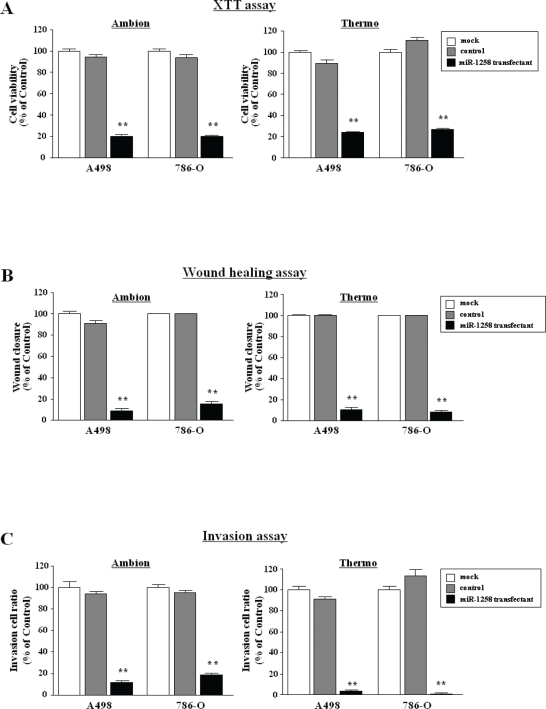
Effect of mature *miR-1285* transfection in RCC cell lines (A) Cell proliferation was determined with XTT assays of A498 and 768-O cell lines after 72 h transfection with 10 nM *miR-1285*, miR-control and mock. Cell proliferation was significantly inhibited in *miR-1285* transfectants in comparison with the mock cells. Thus, with the Ambion products, the percentage of cell viability relative to mock cells was 20.3 ± 1.7% for A498, and 20.1 ± 0.7% for 786-O (both P < 0.0001). For the Thermo products, the percentages were 24.1 ± 1.1% (A498) and 27.1 ± 1.3% (786-O)(both P < 0.0001) (Figure 2A). (B) Cell migration activity was determined with the wound healing assay after 48 h transfection of *miR-1285*. Cell migration was significantly inhibited in *miR-1285* transfectants in comparison with the mock. Thus, with the Ambion materials, the percentage of wound closure relative to mock was 9 ± 6.7% for A498, and 15.4 ± 7.0 for 786-O (both, P < 0.0001). With the Thermo miRNAs, the values were 10.4 ± 6.0% (A498), and 8.2 ± 3.6% (786-O) (both P < 0.0001) (Figure 2B). (C) Cell invasion activity was determined with the Matrigel invasion assay after 48 h transfection of *miR-1285*. Cell numbers significantly decreased after *miR-1285* transfection in comparison with the mock. Thus, using Ambion miRNA, the percentage of cell invasion relative to mock was 11.7 ± 0.7 (A498) and 18.6 ± 3.2% (786-O)(both P < 0.0001). With the Thermo products, we observed 3.1 ± 0.5% (A498)and 0.4 ± 0.7% (786-O) (both P < 0.0001) (Figure 2C). **P < 0.0001.

The XTT assay demonstrated that cell proliferation was significantly inhibited in *miR-1285* transfectants in comparison with the mock cells (Figure 2A).

The wound healing assay demonstrated that cell migration was significantly inhibited in *miR-1285* transfectants in comparison with the mock (Figure 2B).

The Matrigel invasion assay demonstrated that invading cell numbers significantly decreased after *miR-1285* transfection in comparison with the mock (Figure 2C).

### Identification of *miR-1285* regulated target genes by genome-wide gene expression analysis and validation of target genes using clinical RCC specimens

To gain further insight into which genes were affected by *miR-1285* transfection, we performed microarray analysis of *miR-1285* transfectants (A498 and 786-O). A total of 17 genes were downregulated (less than -2.0-fold changes) in *miR-1285* transfectants compared with the controls. The TargetScan program revealed that seven of 17 downregulated genes had putative target sites of *miR-1285* in their 3'UTRs (Table 2).

**Table 2:** Down-regulated genes in miR-1285 transfectants

		Fold change (log 2 ratio)				
Entrez gene ID	Symbol	A498	786-O	Average	Gene name	Target site
**64077**	LHPP	−2.98	−3.92	−3.45	Phospholysine phosphohistidine inorganic pyrophosphate phosphatase	**+**
**4771**	NF2	−2.99	−3.02	−3.01	Neurofibromin 2	**+**
**1979**	EIF4EBP2	−2.79	−3.11	−2.95	Eukaryotic translation initiation factor 4E binding protein 2	**−**
**7052**	TGM2	−2.44	−3.12	−2.78	Transglutaminase 2	**+**
**114902**	C1QTNF5	−2.64	−2.83	−2.74	C1q and tumor necrosis factor related protein 5	**−**
**3773**	KCNJ16	−2.45	−3.00	−2.72	Potassium inwardly-rectifying channel, subfamily J, member 16	**+**
**54901**	CDKAL1	−2.96	−2.31	−2.64	CDK5 regulatory subunit associated protein 1-like 1	**−**
**51148**	CERCAM	−2.52	−2.61	−2.56	Cerebral endothelial cell adhesion molecule	**+**
**11346**	SYNPO	−2.41	−2.58	−2.49	Synaptopodin	**+**
**441518**	FAM127C	−2.09	−2.67	−2.38	Family with sequence similarity 127, member C	**+**
**254439**	C11orf86	−2.41	−2.29	−2.35	Chromosome 11 open reading frame 86	**−**
**83742**	MARVELD1	−2.26	−2.41	−2.34	MARVEL domain containing 1	**−**
**9718**	ECE2	−2.14	−2.50	−2.32	Endothelin converting enzyme 2	**−**
**4907**	NT5E	−2.27	−2.31	−2.29	5'-nucleotidase, ecto (CD73)	**+**
**11313**	LYPLA2	−2.09	−2.31	−2.19	Lysophospholipase II	**+**
**79026**	AHNAK	−2.07	−2.26	−2.16	AHNAK nucleoprotein	**+**
**51313**	FAM198B	−2.03	−2.11	−2.07	Family with sequence similarity 198, member B	**+**

**Figure 3:**
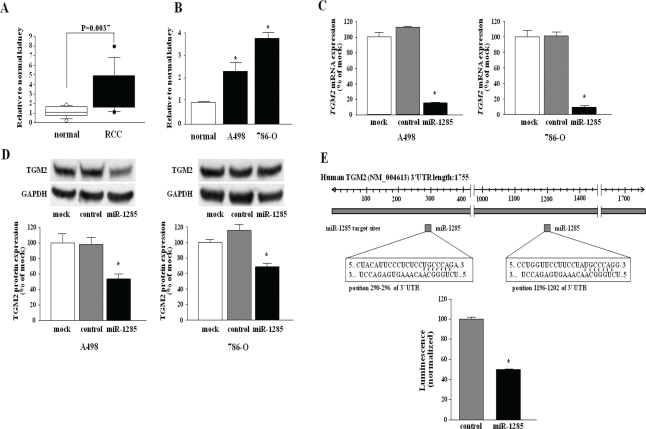
*miR-1285* directly regulates *TGM2* in RCC cells (A) Expression level of *TGM2* mRNA in clinical RCC cell specimens. Relative *TGM2* mRNA expression levels are expressed in box plots. (B) The mRNA expression levels of *TGM2* in RCC cell lines (A498 and 786-O) compared to normal kidney RNA. *GUSB* was used as an internal control. (C) TGM2 mRNA expression in RCC cell lines (A498 and 786-O). *TGM2* mRNA expression 24 h after transfection with 10 nM *miR-1285*. *GUSB* was used as an internal control. (D) TGM2 protein expression in RCC cell lines (A498 and 786-O). TGM2 protein expression 72 h after transfection with 10 nM *miR-1285*. GAPDH was used as a loading control. E) *miRNA-1285* binding sites in the 3'UTR of *TGM2* mRNA. A luciferase assay using the vector encoding full-length 3'UTR of *TGM2* mRNA. The *Renilla* luciferase values were normalized to firefly luciferase values. *P < 0.0001

Seven of the downregulated genes in *miR1285*-transfectants (*LHPP*, *TGM2*, *NF2*, *CERCAM*, *SYNPO*, *LYPLA2*, and *AHNAK*) were selected and we measured the mRNA expression levels in the clinical RCC samples by quantitative real-time RT-PCR. Among them, *TGM2* was the only gene that was expressed significantly higher in RCC specimens than in adjacent non-cancerous tissues (P < 0.0037, Figure 3A). Therefore, we focused on *TGM2* as a promising candidate target of *miR-1285*.

### *miR-1285* directly regulates *TGM2* in RCC cell lines

Quantitative real-time RT-PCR analyses showed that mRNA expression levels of *TGM2* in the A498 and 786-O cell lines were higher than those in normal human kidney (Figure 3B). Furthermore, both *TGM2* mRNA and TGM2 protein expression levels were markedly downregulated in *miR-1285* transfectants in comparison with the control transfectants (A498 and 786-O) (Figure 3C, D).

To determine whether the 3'-UTR of *TGM2* had an actual target site for *miR-1285*, we performed a luciferase reporter assay by using a vector encoding the full-length 3'UTR of *TGM2* mRNA and found that the luminescence intensity was significantly reduced in the *miR-1285* transfectants compared to the control-transfectant (Figure 3E).

### Effect of *TGM2* silencing on cell proliferation, migration and invasion in RCC cell lines

**Figure 4:**
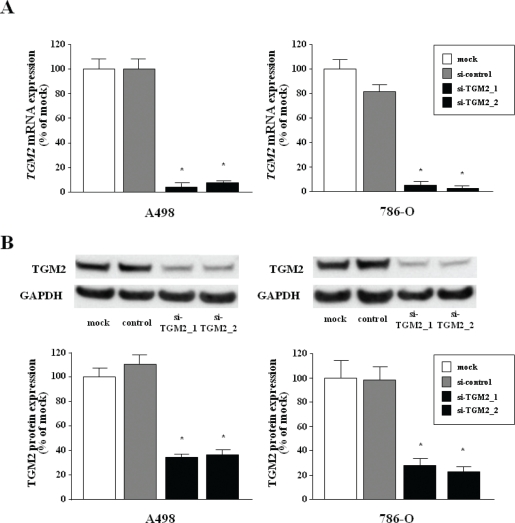
Silencing of *TGM2* in two RCC cell lines by si-*TGM2* (A) *TGM2* mRNA expression after 24 hr of transfection with 10 nM si-*TGM2* in RCC cell lines (A498 and 786-O). *GUSB* was used as an internal control. (B) TGM2 protein expression after 72 hr transfection with si-*TGM2*. GAPDH was used a loading control. *P < 0.0001.

To examine the functional role of *TGM2*, we performed loss-of-function studies in A498 and 786-O cell lines transfected with two different sequences of si-*TGM2*. The mRNA and protein expression levels of TGM2 were markedly repressed by these si-TGM2 transfections (Figure 4A, B).

**Figure 5:**
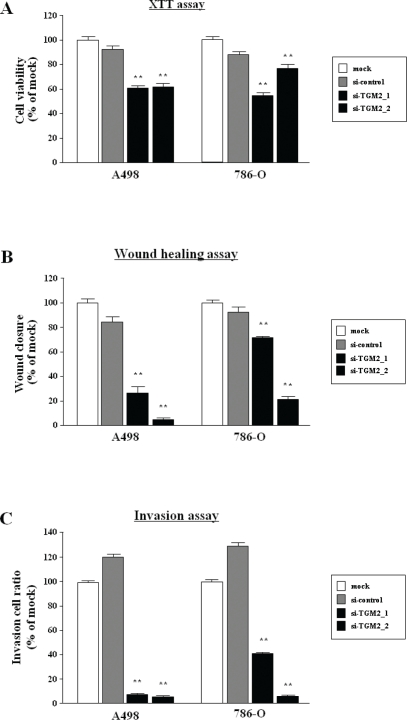
Effect of silencing of *TGM2* in two RCC cell lines (A) Cell proliferation determined with XTT assays of A498 and 768-O cell lines after 72 h transfection with 10nM si-*TRM2*, miR-control or mock. Cell proliferation was significantly inhibited in the two si-*TGM2* transfectants in comparison with the nontransfectants (mock) and the si-control transfectants. Thus, the percentage of cell viability for A498 was 60.6 ± 4.2%, 62.0 ± 5.9%, 100.0 ± 5.5%, and 92.3 ± 6.3%, respectively (P < 0.0001). The percentage of cell viability for 786-O was 54.7 ± 4.7%, 76.9 ± 17.9%, 100.0 ± 6.7% and 88.1 ± 5.5%, respectively, (P < 0.0001) (Figure 5A). (B) Cell migration activity determined with wound healing assays after 48 h transfection with si-*TGM2*. Cell migration was significantly inhibited in the two si-*TGM2* transfectants compared with the counterparts. For A498, the percentage of wound closure was 26.3 ± 14.4%, 4.1 ± 3.9%, 100.0 ± 9.4%, and 84.3 ± 13.0%, respectively (P < 0.0001). For 786-O, we observed closures of 71.2 ± 4.9%, 20.8 ± 6.4%, 100.0 ± 7.5%, and 92.7 ± 13.3%, respectively (P < 0.0001) (Figure 5B). (C) Cell invasion activity determined with the Matrigel invasion assay after 48 h transfection with si-*TGM2*. The number of invading cells was significantly decreased in the two si-*TGM2* transfectants compared with the counterparts. Specifically, the percentage of cell invasion for A498 was 7.3 ± 2.6%, 5.1 ± 1.9%, 100.0 ± 4.0%, and 120.7 ± 4.0%, respectively (P < 0.0001) and for 786-O, they were 40.7 ± 3.8%, 55.0 ± 51.2%, 100.0 ± 3.7%, and 136.4 ± 8.1%, respectively (P < 0.0001) (Figure 5C).**P < 0.0001.

The XTT assay revealed that significant inhibition of cell proliferation was observed in the two si-*TGM2* transfectants in comparison with the untransfectants (mock) and the si-control transfectants (Figure 5A).

The wound healing assay also demonstrated significant cell migration inhibitions in the two si-*TGM2* transfectants compared with the counterparts (Figure 5B).

The matrigel invasion assay demonstrated that the number of invading cell was significantly decreased in the two si-*TGM2* transfectants compared with the counterparts (Figure 5C).

### Immunohistochemistry of *TGM2*

**Figure 6:**
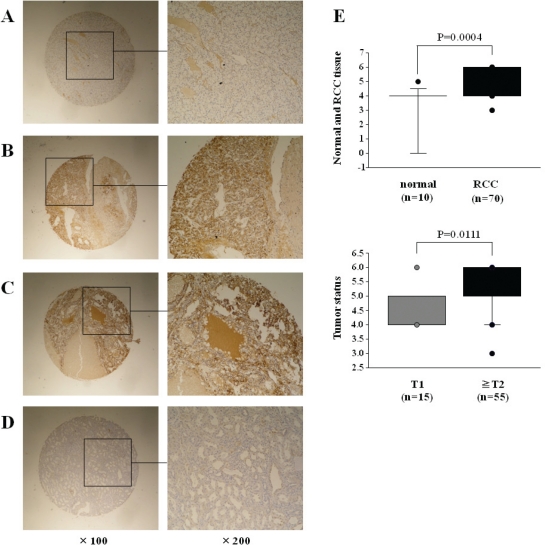
Immunohistochemical staining of *TGM2* in tissue specimens (A) Positively stained tumor lesion (Grade 1, T1N0M0); (B) positively stained tumor lesion (Grade 1, T2N0M0); (C) positively stained tumor lesion (Grade 1, T3N0M0); (D) Negative staining in normal urocustis tissue. (A, B, C) positive staining in tumor cells: weak (A), strong (B, C). (E) TGM2 expression levels in immunohistochemical staining; upper, TGM2 expression in normal kidney and RCC; lower, correlation between TGM2 expression and clinicopathological parameters in RCC.

Figure 6 shows the representative results of immunohistochemical staining of TGM2. TGM2 was strongly expressed in tumor lesions A (T1N0M0), B (T2N0M0) and C (T3N0M0), whereas no expression was observed in normal tissue (D). The expression score of the tumor was significantly higher than that of normal tissues (P = 0.0004) (E, upper). We found that there were significant correlations between the expression scores and tumor stage (P = 0.0111) (E, lower).

## DISCUSSION

In this study, we constructed miRNA expression signatures of clinical RCC specimens using 778 miRNAs by PCR-based analysis. Among them, 103 miRNAs were downregulated (0.5-fold change) and the top 20 downregulated miRNAs were evaluated to determine whether these miRNAs had potential tumor suppressor functions. Our functional screening revealed that 14 miRNAs (*miR-1285*, *miR-206*, *miR-1*, *miR-135a*, *miR-429*, *miR-200c*, *mR-1291*, *miR-133b*, *miR-508-3p*, *miR-362-3p*, *miR-509-5p*, *miR-218*, *miR-335*, *miR-1255b* and *miR-141*) significantly inhibited cancer cell proliferation in RCC cell lines, suggesting that these miRNAs were potentially promising candidate tumor suppressive miRNAs.

In our previous studies of miRNA signatures, *miR-206*, *miR-1*, *miR-133b* and *miR-218* were significantly reduced in various types of cancers. We also observed that restoration of these miRNAs inhibited cancer cell proliferation, invasion and migration [10-16]. Our data strongly suggested that these miRNAs function as tumor suppressors in human cancers [10-16]. Of particular interest, *miR-1-1/miR-133a-2*, *miR-1-2/miR-133a-1*, and *miR-206/miR-133b* form clusters on three different chromosomal regions in the human genome, 20q13.33, 18q11.2, and 6p12.1, respectively [17, 18]. We and other groups also demonstrated that these clusters were frequently downregulated in human cancers and significantly contributed to human oncogenesis [8, 10, 19-22]. Our present signature of RCC supports previous results and it is expected that these function as tumor suppressors in RCC.

In this signature, *miR-141*, *miR-200c*, *miR-429*, *miR-200b* and *miR-200a* were significantly reduced in RCC. It is well known that the *miR-200* family consists of five members organized as two clusters, *miR-200b/miR-200a/miR-429* and *miR-200c/miR-141*, on chromosomes 1p36.33 and 12p13.31, respectively. The miR-200 family contains closely related seed sequences. It has been reported that one can inhibit the initiation of the epithelial-mesenchymal transition (EMT) by targeting *ZEB1* and *ZEB2* [23, 24]. Downregulation of the *miR-200* family in our signature is supported by other reports of RCC signatures [25], and this fact suggests that the EMT pathway is a main theme of RCC oncogenesis. Our miRNA expression signature provides the important information for miRNA research fields of RCC oncogenesis and metastasis.

In this study, we focused on *miR-1285* because it had the greatest inhibitory effect on cancer cell proliferation among 20 downregulated miRNAs in our signature. We investigated its functional significance and how it regulated molecular targets in RCC cells. *miR-1285* was discovered from massive parallel sequencing of human embryonic stem cells [26], and it is a unique miRNA that exists in a limited group of animals including *Homo sapiens*, *Pan troglodytes*, *Sus scrofa* and *Pongo pygmaeus* (miRBase: http://www.mirbase.org/index.shtml). In the human genome, *miR-1285* mapped on two different chromosomes (*miR-1285-1* at 7q21.2 and *miR-1285-2* at 2p13.3), and the mature miRNA sequences are identical. There are few publications focused on *miR-1285*. This is the first report that *miR-1285* is downregulated in clinical RCC specimens and demonstrates that it functions as a tumor suppressor.

miRNAs are unique in their ability to regulate many protein coding genes. Bioinformatic predictions indicate that miRNAs regulate more than 30% of protein coding genes [27]. The elucidation of new molecular targets regulated by tumor suppressive *miR-1285* is important for our understanding of RCC oncogenesis. Based on this view, we have performed to search miR-1285 regulated molecular targets by using genome-wide gene expression analysis.

In this study, we identified seven target genes (*LHPP*, *TGM2*, *NF2*, *CERCAM*, *SYNPO*, *LYPLA2*, and *AHNAK*) downregulated in *miR-1285*-transfected cells and found they contained *miR-1285* target sites in their 3'-UTR. Next, we investigated the mRNA expression levels of seven candidate genes using clinical RCC specimens. *TGM* was the most upregulated gene in cancer cells. Thus, we examined the role of *TGM2* in RCC cells. TGM2 is a family of enzymes that catalyzes the formation of an amide bond between the γ-carboxamide groups of peptide-bound glutamine residues and the primary amino group in various compounds [28, 29]. *TGM2* is known to play an important role in cancer. Increased expression of *TGM2* has been observed in many types of cancer, including pancreatic cancer [30], breast cancer [31], malignant melanoma [32], ovarian cancer [33], lung cancer [34], and glioblastoma [35]. In addition, several investigators showed that increased expression of *TGM2* might be linked to increased drug resistance, metastasis, and the epithelial to mesenchymal transition (EMT) [36-39]. Our present data support reports finding that *TGM2* functions as an oncogene in RCC.

In conclusion, *miR-1285* was significantly downregulated in RCC cell lines, was frequently reduced in clinical specimens, and functioned as a tumor suppressor in RCC. Our data indicated that upregulation of oncogenic *TGM2* may be due to downregulation of tumor suppressive *miR-1285* in human RCC progression. This novel molecular network may play a critical role in RCC oncogenesis and serve as a novel therapeutic strategy for patients with RCC.

## METHODS

### RCC Clinical specimens and RCC cell lines

Following nephrectomies at Kagoshima University Hospital, a total of five pairs of clear cell type cancer and adjacent non-cancerous tissue, and an additional group of five clear cell type cancer were collected for miRNA expression analysis (supplementary Table 3, Number 1-15). The tissue specimens for quantitative RT-PCR were from 36 RCC patients who had undergone nephrectomy at Kagoshima University Hospital between 2006 and 2009 (supplementary Table 3, Numbers 6 - 43). These samples were staged according to the American Joint Committee on Cancer-Union Internationale Contre le Cancer (UICC) tumor-node-metastasis classification and histologically graded [40]. Our study was approved by the Bioethics Committee of Kagoshima University; written prior informed consent and approval were given by the patients.

We used four human RCC cell lines: A498, 786-O, ACHN and caki-2 that were obtained from the American Type Culture Collection (Manassas, VA, USA). These cell lines were incubated in RPMI 1640 medium (Invitrogen, Carlsbad, CA, USA) supplemented with 10% fetal bovine serum and maintained in humidified incubators (5% CO2) at 37°C.

Total RNA including miRNA was extracted using the mirVana miRNA isolation kit (Ambion, Austin, TX, USA) following the manufacturer's protocol. The integrity of the RNA was checked with the RNA 6000 Nano Assay Kit and a 2100 Bioanalyzer (Agilent Technologies, Santa Clara, CA, USA).

### miRNA expression signatures and data normalization

MiRNA expression patterns were evaluated using the TaqMan LDA Human microRNA Panel v2.0; a total of 778 miRNAs were investigated in the screen (Applied Biosystems, Foster City, CA, USA). The assay was composed of two steps: generation of cDNAs by reverse transcription and a TaqMan real-time PCR assay. The description of real-time PCR and the list of human miRNAs can be found on the company's website (http://www.appliedbiosystems.com). An analysis of relative miRNA expression data was performed using GeneSpring GX version 7.3.1 software (Agilent Technologies) according to the manufacturer's instructions. A cutoff P value < 0.05 was used to narrow down the candidates after global normalization of the raw data. After global normalization, the additional normalization was done with *RNU48*.

### Quantitative real-time RT-PCR

TaqMan probes and primers for *TGM2* (P/N: Hs00190278_m1; Applied Biosystems) were assay-on-demand gene expression products. All reactions were performed in duplicate and a negative control lacking cDNA was included. We followed the manufacturer's protocol for PCR conditions. Stem-loop RT–PCR (TaqMan MicroRNA Assays; P/N: PM13580 for *miR-1285*; Applied Biosystems) was used to quantitate miRNAs according to earlier published conditions (11). To normalize the data for quantification of *TGM2* mRNA and the miRNAs, we used *human GUSB* (P/N: Hs99999908_m1; Applied Biosystems) and *RNU48* (P/N: 001006; Applied Biosystems), respectively, and the delta–delta Ct method was employed to calculate the fold-change. As a control RNA, we used total RNA from our normal human kidneys sample.

### Mature miRNA and siRNA transfection

As described elsewhere (11), the RCC cell lines were transfected with Lipofectamine RNAiMAX transfection reagent (Invitrogen) and Opti-MEM (Invitrogen) with 10 nM mature miRNA molecules. Pre-miR (Applied Biosystems and Thermo Fisher Scientific) and negative control miRNA (Applied Biosystems) were used in the gain-of-function experiments, whereas *TGM2* siRNA (Cat numbers, SASI_Hs01_00035266 and SASI_Hs02_00338000, Sigma Aldrich) and negative control siRNA (MISSION siRNA Universal Negative Control, Sigma Aldrich) were used in the loss-of-function experiments. Cells were seeded in ten cm dishes for protein extraction (8 × 10^5^ cells per dish), in six well plates for wound healing assays (20 × 10^4^ cells per well), in a 24 well plates for mRNA extraction and luciferase reporter assays (5 × 10^4^ cells per well), and in 96 well plates for XTT assays (3000 cells per well).

### Cell proliferation, migration and invasion assays

Cell proliferation was determined using an XTT assay (Roche Applied Sciences, Tokyo, Japan) performed according to the manufacturer's instructions. Cell migration activity was evaluated with a wound-healing assay. Cells were plated in six well dishes, and the cell monolayer was scraped using a P-20 micropipette tip. The initial gap length (0 h) and the residual gap length 24 h after wounding were calculated from photomicrographs. A cell invasion assay was carried out using modified Boyden Chambers consisting of Transwell-precoated Matrigel membrane filter inserts with eight mm pores in 24 well tissue culture plates (BD Biosciences, Bedford, MA, USA). Minimum essential medium containing 10% fetal bovine serum in the lower chamber served as the chemoattractant as described previously (12). All experiments were performed in triplicate.

### Screening of *miR-1285*-regulated genes using microarray and database analysis

Oligo-microarray Human 44K (Agilent) was used for expression profiling in *miR-1285*-transfected RCC cell lines (A498 and 786-O) in comparison with miR-negative control transfectants, as previously described (12). Briefly, hybridization and washing steps were performed in accordance with the manufacturer's instructions. The arrays were scanned using a Packard GSI Lumonics ScanArray 4000 (PerkinElmer, Boston, MA, USA). The data obtained were analyzed with DNASIS array software (Hitachi Software Engineering, Tokyo, Japan), which converted the signal intensity for each spot into text format. The log_2_ ratios of the median subtracted background intensity were analyzed. Data from each microarray study were normalized by global normalization.

The predicted target genes and their miRNA binding site seed regions were investigated using TargetScan (release 5.1, http://www.targetscan.org/). The sequences of the predicted mature miRNAs were confirmed using miRBase (release 18.0, November 2011; http://microrna.sanger.ac.uk/).

### Western blots

After three days of transfection, protein lysates (20 μg) were separated by NuPAGE on 4–12% bis-tris gels (Invitrogen) and transferred to polyvinylidene fluoride membranes. Immunoblotting was done with diluted (1:200) polyclonal TGM2 antibody (HPA029518; Sigma-Aldrich, St. Louis, MO, USA) and GAPDH antibody (MAB374; Chemicon, Temecula, CA, USA). The membrane was washed and then incubated with goat anti-rabbit IgG (H+L)-HRP conjugate (BIO-RAD, Hercules, CA, USA). Specific complexes were visualized with an echochemiluminescence (ECL) detection system (GE Healthcare, Little Chalfont, UK), and the expression levels of these genes were evaluated using ImageJ software (ver. 1.43; http://rsbweb.nih.gov/ij/index.html).

### Plasmid construction and dual-luciferase reporter assay

The miRNA target sequences were inserted between the *Xho*I–*Pme*I restriction sites in the 3'-UTR of the *hRluc* gene in the psiCHECK-2 vector (C8021; Promega, Madison, WI, USA). Primer sequences for the full-length 3'UTR of *TGM2* mRNA (5'-GATCGCTCGAGCCACCTTGATTCCCAATCC-3' and5'-CTTAAACTGTGACTCTTTCCTGTGCAA-3') were designed. 786-O cells were transfected with 15 ng vector, 10 nM microRNAs, and one μL Lipofectamine 2000 (Invitrogen) in 100 μL Opti-MEM (Invitrogen). The activities of firefly and *Renilla* luciferases in cell lysates were determined with a dual-luciferase assay system (E1910; Promega). Normalized data were calculated as the quotient of *Renilla*/firefly luciferase activities.

### Immunohistochemistry

A tissue microarray of 70 renal cell carcinomas and ten normal kidneys was obtained from US Biomax, Inc. (KD806; Rockville, MD, USA). Detailed information on all tumor specimens can be found at http://www.biomax.us/index.php. The patients' backgrounds and clinicopathological characteristics are summarized in supplementary Table 4. The tissue microarray was immunostained following the manufacturer's protocol with an UltraVision Detection System (Thermo Scientific). The primary rabbit polyclonal antibodies against TGM2 (Sigma-Aldrich) were diluted 1:400. The slides were treated with biotinylated goat anti-rabbit antibodies. Diaminobenzidine hydrogen peroxidase was the chromogen, and counterstaining was done with 0.5% hematoxylin. Immunostaining was evaluated according to a scoring method described previously [13]. Each case was scored on the basis of the intensity and area of staining. The intensity of staining was graded on the following scale: 0, no staining; 1+, mild staining; 2+, moderate staining; and 3+, intense staining. The area of staining was evaluated as follows: 0, no staining of cells in any microscopic fields; 1+, < 30% of cells stained positive; 2+, 30–60% stained positive; 3+, > 60% stained positive. A combined staining score (intensity + extension) of < 2 was low expression, a score between 3 and 4 was moderate expression, and a score between 5 and 6 was high expression.

### Statistical analysis

The relationships between two variables and the numerical values obtained by real-time RT-PCR were analyzed using the Mann-Whitney U test. The relationships among three variables and the numerical values were analyzed using the Bonferroni-adjusted Mann-Whitney U test. The χ^2^-test was used to evaluate the relationships between immunohistochemical scores of TGM2 expression and clinicopathological factors. Expert StatView analysis software (version 4; SAS Institute Inc., Cary, NC, USA) was used in both cases. In the comparison among three variables, a nonadjusted statistical level of significance of P < 0.05 corresponds to a Bonferroni-adjusted level of P < 0.0167.

## Supplementary Tables








