# Supplementary material for: Tumor suppressive microRNA-1285 regulates novel molecular targets: Aberrant expression and functional significance in renal cell carcinoma
Source: Oncotarget. 2012 Jan 30;3(1):44–57. doi: 10.18632/oncotarget.417 (PMC3292891; doi:10.18632/oncotarget.417)
Supplement: Supplementary file 1 [file oncotarget-03-044-s001.docx]

|  |  |  |  |  |
| --- | --- | --- | --- | --- |
| **Table S1: Down-regulated microRNAs in renal cell carcinoma (RCC)** | | | | |
| **microRNA** | **P-value** | **Normal** | **Cancer** | **Fold Change** |
|  |  |  |  | **(Cancer/Normal)** |
| hsa-miR-141 | 0.022 | 1.237 | 0.026 | 0.021 |
| hsa-miR-200c | 0.022 | 1.104 | 0.024 | 0.021 |
| hsa-miR-187 | 0.007 | 1.526 | 0.043 | 0.028 |
| hsa-miR-509-5p | 0.003 | 1.196 | 0.050 | 0.042 |
| hsa-miR-135a | 0.003 | 1.525 | 0.099 | 0.065 |
| hsa-miR-508-3p | 0.007 | 1.321 | 0.096 | 0.072 |
| hsa-miR-1285 | 0.020 | 1.777 | 0.171 | 0.096 |
| hsa-miR-206 | 0.013 | 1.580 | 0.192 | 0.121 |
| hsa-miR-218 | 0.005 | 1.506 | 0.197 | 0.130 |
| hsa-miR-133b | 0.006 | 1.173 | 0.173 | 0.147 |
| hsa-miR-1291 | 0.019 | 1.978 | 0.310 | 0.157 |
| hsa-let-7g* | 0.031 | 1.508 | 0.247 | 0.164 |
| hsa-miR-204 | 0.014 | 1.468 | 0.254 | 0.173 |
| hsa-miR-429 | 0.003 | 1.267 | 0.222 | 0.175 |
| hsa-miR-370 | 0.042 | 1.525 | 0.268 | 0.176 |
| hsa-miR-363 | 0.010 | 1.377 | 0.244 | 0.177 |
| hsa-miR-335 | 0.005 | 1.226 | 0.224 | 0.182 |
| hsa-miR-1 | 0.005 | 1.017 | 0.189 | 0.186 |
| hsa-miR-1255B | 0.020 | 1.306 | 0.248 | 0.190 |
| hsa-miR-362-3p | 0.010 | 1.501 | 0.312 | 0.208 |
| hsa-miR-200b | 0.003 | 1.186 | 0.249 | 0.210 |
| hsa-miR-138 | 0.020 | 1.306 | 0.277 | 0.212 |
| hsa-miR-10b | 0.031 | 1.348 | 0.292 | 0.217 |
| hsa-miR-660 | 0.003 | 1.276 | 0.283 | 0.222 |
| hsa-miR-214* | 0.010 | 1.232 | 0.283 | 0.230 |
| hsa-miR-655 | 0.007 | 1.216 | 0.280 | 0.231 |
| hsa-miR-9 | 0.014 | 1.336 | 0.320 | 0.240 |
| hsa-miR-133a | 0.014 | 1.182 | 0.287 | 0.243 |
| hsa-miR-532-5p | 0.002 | 1.179 | 0.289 | 0.245 |
| hsa-miR-26a-1 | 0.003 | 1.114 | 0.278 | 0.249 |
| hsa-miR-432 | 0.013 | 1.075 | 0.269 | 0.250 |
| hsa-miR-200a | 0.010 | 1.240 | 0.313 | 0.252 |
| hsa-miR-10a | 0.005 | 1.180 | 0.302 | 0.256 |
| hsa-miR-30a-3p | 0.005 | 1.192 | 0.307 | 0.258 |
| hsa-miR-136* | 0.010 | 1.120 | 0.289 | 0.258 |
| hsa-miR-127-3p | 0.005 | 1.151 | 0.297 | 0.258 |
| hsa-miR-135b | 0.014 | 1.210 | 0.315 | 0.260 |
| hsa-miR-30d* | 0.003 | 1.242 | 0.326 | 0.263 |
| hsa-miR-192* | 0.066 | 1.614 | 0.425 | 0.263 |
| hsa-miR-501-5p | 0.007 | 1.230 | 0.326 | 0.265 |
| hsa-miR-194 | 0.111 | 1.756 | 0.468 | 0.266 |
| hsa-miR-376c | 0.005 | 1.113 | 0.305 | 0.274 |
| hsa-miR-149 | 0.014 | 1.124 | 0.308 | 0.274 |
| hsa-miR-23b | 0.005 | 1.209 | 0.332 | 0.275 |
| hsa-miR-191* | 0.464 | 2.168 | 0.599 | 0.277 |
| hsa-miR-30c | 0.010 | 1.218 | 0.337 | 0.277 |
| hsa-miR-337-5p | 0.019 | 1.272 | 0.354 | 0.278 |
| hsa-miR-214 | 0.005 | 1.108 | 0.309 | 0.279 |
| hsa-miR-199a-3p | 0.014 | 1.193 | 0.334 | 0.280 |
| hsa-miR-200a* | 0.014 | 1.293 | 0.363 | 0.281 |
| hsa-miR-30e-3p | 0.003 | 1.158 | 0.327 | 0.283 |
| hsa-miR-203 | 0.020 | 1.204 | 0.342 | 0.284 |
| hsa-miR-190 | 0.086 | 1.572 | 0.447 | 0.284 |
| hsa-miR-30a-5p | 0.010 | 1.249 | 0.364 | 0.292 |
| hsa-miR-335* | 0.020 | 1.188 | 0.354 | 0.298 |
| hsa-miR-27b* | 0.028 | 1.380 | 0.412 | 0.299 |
| hsa-miR-378 | 0.111 | 1.594 | 0.477 | 0.299 |
| hsa-miR-331-5p | 0.205 | 2.249 | 0.675 | 0.300 |
| hsa-miR-125a-3p | 0.037 | 1.285 | 0.400 | 0.311 |
| hsa-miR-30b | 0.010 | 1.219 | 0.387 | 0.317 |
| hsa-miR-500 | 0.005 | 1.221 | 0.390 | 0.319 |
| hsa-miR-199a-5p | 0.020 | 1.173 | 0.384 | 0.327 |
| hsa-miR-411 | 0.028 | 1.219 | 0.401 | 0.329 |
| hsa-miR-454* | 0.066 | 1.353 | 0.460 | 0.340 |
| hsa-miR-9* | 0.028 | 1.189 | 0.407 | 0.342 |
| hsa-miR-425* | 0.010 | 1.158 | 0.398 | 0.344 |
| hsa-miR-422a | 0.066 | 1.414 | 0.490 | 0.346 |
| hsa-miR-502-3p | 0.020 | 1.184 | 0.425 | 0.359 |
| hsa-miR-379 | 0.042 | 1.249 | 0.453 | 0.363 |
| hsa-miR-33a* | 0.009 | 1.065 | 0.395 | 0.371 |
| hsa-miR-100 | 0.037 | 1.181 | 0.441 | 0.374 |
| hsa-miR-338-3p | 0.739 | 2.544 | 0.953 | 0.375 |
| hsa-miR-125b | 0.005 | 1.121 | 0.424 | 0.379 |
| hsa-miR-487b | 0.028 | 1.149 | 0.437 | 0.381 |
| hsa-miR-192 | 0.178 | 1.629 | 0.632 | 0.388 |
| hsa-miR-10a* | 0.013 | 1.063 | 0.413 | 0.389 |
| hsa-miR-99a | 0.028 | 1.127 | 0.441 | 0.391 |
| hsa-miR-20b | 0.037 | 1.224 | 0.478 | 0.391 |
| hsa-miR-139-5p | 0.028 | 1.140 | 0.447 | 0.392 |
| hsa-miR-27b | 0.113 | 1.283 | 0.507 | 0.395 |
| hsa-miR-539 | 0.014 | 1.117 | 0.445 | 0.398 |
| hsa-miR-664 | 0.010 | 1.204 | 0.480 | 0.399 |
| hsa-miR-495 | 0.050 | 1.347 | 0.542 | 0.403 |
| rno-miR-29c* | 0.066 | 1.268 | 0.529 | 0.417 |
| hsa-miR-378 | 0.142 | 1.421 | 0.604 | 0.425 |
| hsa-miR-26b* | 0.028 | 1.117 | 0.476 | 0.426 |
| hsa-miR-638 | 0.066 | 1.652 | 0.706 | 0.427 |
| hsa-let-7g | 0.020 | 1.192 | 0.512 | 0.430 |
| hsa-miR-502-5p | 0.050 | 1.197 | 0.521 | 0.435 |
| hsa-miR-125a-5p | 0.005 | 1.116 | 0.491 | 0.440 |
| hsa-miR-99b* | 0.037 | 1.086 | 0.482 | 0.443 |
| hsa-miR-30d | 0.005 | 1.155 | 0.515 | 0.446 |
| hsa-miR-598 | 0.037 | 1.179 | 0.535 | 0.454 |
| hsa-miR-134 | 0.050 | 1.488 | 0.682 | 0.459 |
| hsa-miR-542-5p | 0.096 | 1.493 | 0.685 | 0.459 |
| hsa-miR-375 | 0.088 | 1.154 | 0.536 | 0.464 |
| hsa-miR-362-5p | 0.005 | 1.098 | 0.511 | 0.465 |
| hsa-miR-376a | 0.050 | 1.145 | 0.546 | 0.477 |
| hsa-miR-99a* | 0.050 | 1.172 | 0.559 | 0.477 |
| hsa-miR-485-3p | 0.807 | 2.549 | 1.228 | 0.482 |
| hsa-miR-656 | 0.464 | 1.445 | 0.722 | 0.500 |
