# Supplementary material for: Tumor suppressive microRNA-1285 regulates novel molecular targets: Aberrant expression and functional significance in renal cell carcinoma
Source: Oncotarget. 2012 Jan 30;3(1):44–57. doi: 10.18632/oncotarget.417 (PMC3292891; doi:10.18632/oncotarget.417)
Supplement: Supplementary file 2 [file oncotarget-03-044-s002.docx]

| **Table S2: Cell proliferation inhibition rate (relative to mock)** | | |
| --- | --- | --- |
|  |  |  |
|  | **miRNA** | **%** |
| 1 | miR-1285 | 50.3 |
| 2 | miR-206 | 58.2 |
| 3 | miR-1 | 60.4 |
| 4 | miR-135a | 65.9 |
| 5 | miR-429 | 68.8 |
| 6 | miR-200c | 70.5 |
| 7 | miR-1291 | 72.9 |
| 8 | miR-133b | 73.5 |
| 9 | miR-508-3p | 74.8 |
| 10 | miR-362-3p | 75.5 |
| 11 | miR-509-5p | 77.1 |
| 12 | miR-218 | 77.1 |
| 13 | miR-335 | 79.1 |
| 14 | miR-1255b | 79.4 |
| 15 | miR-141 | 79.7 |
| 16 | miR-204 | 81.5 |
| 17 | miR-let-7g | 85.1 |
| 18 | miR-370 | 86.6 |
| 19 | miR-363 | 89.4 |
| 20 | miR-187 | 90.8 |
