# Supplementary material for: Tumor suppressive microRNA-1285 regulates novel molecular targets: Aberrant expression and functional significance in renal cell carcinoma
Source: Oncotarget. 2012 Jan 30;3(1):44–57. doi: 10.18632/oncotarget.417 (PMC3292891; doi:10.18632/oncotarget.417)
Supplement: Supplementary file 3 [file oncotarget-03-044-s003.docx]

| **Table S3:**  **Patients' characteristics** | | |  |  |  |  |  |
| --- | --- | --- | --- | --- | --- | --- | --- |
| **No** | **Cell type** | **Age** | **Gender** | **Pathological tumor stage** | **Grade** | **Infiltration** | **Venous invasion** |
| 1 | normal |  |  | - | - | - | - |
| 2 | normal |  |  | - | - | - | - |
| 3 | normal |  |  | - | - | - | - |
| 4 | normal |  |  | - | - | - | - |
| 5 | normal |  |  | - | - | - | - |
| 6 | clear cell carcinoma | 42 | male | pT1a | 1 | α | V (-) |
| 7 | clear cell carcinoma | 64 | female | pT1a | 2 | α | V (-) |
| 8 | clear cell carcinoma | 53 | male | pT1a | 2 | α | V (-) |
| 9 | clear cell carcinoma | 77 | male | pT1a | 1 | α | V (-) |
| 10 | clear cell carcinoma | 74 | male | pT1a | 1 | α | V (-) |
| 11 | clear cell carcinoma | 46 | male | pT3b | 2 | β | V (+) |
| 12 | clear cell carcinoma | 69 | male | pT3a | 3 | β | V (+) |
| 13 | clear cell carcinoma | 81 | female | pT4 | 3 | β | V (+) |
| 14 | clear cell carcinoma | 59 | male | pT3a | 2 | β | V (-) |
| 15 | clear cell carcinoma | 78 | male | pT3b | 3 | α | V (+) |
| 16 | clear cell carcinoma | 66 | male | pT1a | 1 | α | V (-) |
| 17 | clear cell carcinoma | 80 | male | pT1a | 2 | α | V (-) |
| 18 | clear cell carcinoma | 46 | female | pT1b | 2 | α | V (+) |
| 19 | clear cell carcinoma | 54 | male | pT1b | 2 | α | V (+) |
| 20 | clear cell carcinoma | 68 | male | pT1b | 2 | α | V (-) |
| 21 | clear cell carcinoma | 54 | male | pT1b | 2 | α | V (-) |
| 22 | clear cell carcinoma | 73 | female | pT1b | 2 | α | V (-) |
| 23 | clear cell carcinoma | 83 | female | pT1a | 1 | α | V (-) |
| 24 | clear cell carcinoma | 64 | male | pT1a | 1 | α | V (-) |
| 25 | clear cell carcinoma | 70 | male | pT1b | 2 | β | V (-) |
| 26 | clear cell carcinoma | 75 | Male | pT1a | 2 | α | V (+) |
| 27 | clear cell carcinoma | 70 | female | pT1a | 2 | α | V (-) |
| 29 | clear cell carcinoma | 69 | male | pT1a | 2 | β | V (-) |
| 30 | clear cell carcinoma | 79 | female | pT1a | unknown | β | V (-) |
| 31 | clear cell carcinoma | 72 | male | pT1b | 2 | α | V (-) |
| 32 | clear cell carcinoma | 62 | male | pT1a | 2 | α | V (-) |
| 33 | clear cell carcinoma | 82 | female | pT3b | 2 | α | V (+) |
| 35 | clear cell carcinoma | 42 | female | pT1b | 2 | β | V (-) |
| 36 | clear cell carcinoma | 36 | female | pT1b | 2 | α | V (-) |
| 37 | clear cell carcinoma | 65 | male | pT1b | 2 | β | V (-) |
| 38 | clear cell carcinoma | 36 | male | pT1a | 2 | α | V (-) |
| 39 | clear cell carcinoma | 62 | male | pT1a | 1 | α | V (-) |
| 40 | clear cell carcinoma | 38 | female | pT2 | 2 | α | V (-) |
| 41 | clear cell carcinoma | 67 | female | pT1a | 2 | α | V (-) |
| 42 | clear cell carcinoma | 68 | male | pT3a | 2 | β | V (+) |
| 43 | clear cell carcinoma | 69 | female | pT2 | 2 | β | V (+) |
