# Supplementary material for: Tumor suppressive microRNA-1285 regulates novel molecular targets: Aberrant expression and functional significance in renal cell carcinoma
Source: Oncotarget. 2012 Jan 30;3(1):44–57. doi: 10.18632/oncotarget.417 (PMC3292891; doi:10.18632/oncotarget.417)
Supplement: Supplementary file 4 [file oncotarget-03-044-s004.docx]

| **Table S4 Patients' characteristics for tissue microarray** | |
| --- | --- |
|  | **n** |
| Total number | 70 |
| Median Age (range) in years | 54 (33-80) |
| Gender |  |
| male | 46 |
| female | 24 |
| Pathological tumor stage |  |
| pT1 | 15 |
| pT2 | 28 |
| pT3 | 25 |
| pT4 | 2 |
| Grade |  |
| G1 | 52 |
| G2 | 14 |
| G3 | 1 |
| unknown | 3 |
